# Morbidity burden and sleep disorder risk among occupational radiation-exposed workers: a cross-sectional study of 1,089 participants in southern China

**DOI:** 10.3389/fpubh.2025.1686994

**Published:** 2026-01-05

**Authors:** Hai-Bo Huang, Yi-Wei Su, Shi-Feng Hou, Yan Zhang, Wan-Feng Zhang, Jian-Wei Liao, Ci-Jian Wu, Zhi Wang, Jian-Yu Wang

**Affiliations:** Key Laboratory of Occupational Environment and Health, Guangzhou Twelfth People's Hospital, Guangzhou, China

**Keywords:** occupational radiation exposure, morbidity burden, sleep disorders, cross-sectional study, China

## Abstract

**Background:**

Occupational radiation exposure poses unique health challenges, with emerging evidence suggesting links between chronic low-dose exposure, multisystem morbidity, and sleep disturbances. This study examines the relationship between cumulative morbidity burden and the risk of sleep disorders among radiation-exposed workers in southern China.

**Methods:**

A cross-sectional investigation was conducted from January to December 2024 at Guangzhou Twelfth People’s Hospital. Morbidity burden was assessed through physician-diagnosed conditions classified by ICD-10 codes across seven disease categories. Sleep disorders were identified via a study-specific questionnaire. Multivariable logistic regression models, adjusted for demographic, occupational, and lifestyle confounders, were used to quantify associations between morbidity and sleep disorders. Subgroup analyses evaluated effect modification by sex, service duration, and profession.

**Results:**

A consecutive sample of 1,089 radiation workers underwent comprehensive health evaluations. Sleep disorders affected 33.0% of participants. A graded dose–response relationship was observed: workers with one morbidity exhibited 2.28-fold higher risk of sleep disorders (95%CI:1.68–3.10) compared to those without comorbidities. Risk increased to OR = 2.89 (1.97–4.25) for two morbidities and OR = 3.81 (2.42–6.01) for ≥3 morbidities after full adjustment. Subgroup analyses revealed significantly stronger associations in women (OR = 3.97, 1.94–8.42), workers with ≤15 years of service (OR = 4.24, 1.97–9.38), and biomedical engineers (OR = 5.75, 2.38–14.33). Thyroid, respiratory, cardiovascular, and lens opacity prevalence differed substantially between workers with sleep disorders and those without.

**Conclusion:**

Accumulating morbidity burden is robustly associated with sleep disorder risk among radiation workers, with occupational factors influencing the strength of this association. Biomedical engineers, women, and early-career personnel represent high-risk subgroups that warrant targeted screening and preventive interventions.

## Introduction

Occupational radiation exposure remains a significant concern across industrial, healthcare, and research sectors, with surveillance data indicating a continually growing monitored workforce in China (1–3). While stringent safety protocols mitigate acute health effects, the long-term implications of chronic low-dose radiation exposure—particularly regarding non-cancer morbidity and its impact on sleep health—require sustained scientific attention (4). Epidemiological studies consistently report elevated risks of endocrine dysfunction in radiation-exposed workers, with thyroid disorders demonstrating a disproportionately higher burden compared to non-exposed groups (4–6). Concurrently, sleep disturbances represent a pervasive occupational health challenge, affecting substantial proportions of shift workers globally (7).

The convergence of radiation exposure, occupational stressors, and environmental factors may disrupt core physiological pathways. Experimental evidence confirms that ionizing radiation can induce circadian misalignment at the molecular level in human peripheral cells (8). Crucially, the suprachiasmatic nucleus exhibits heightened vulnerability to environmental perturbations that compromise neuronal synchrony (9). Radiation exposure further dysregulates hypothalamic orexinergic pathways essential for sleep–wake regulation, as evidenced by measurable alterations in sleep architecture (10). These disruptions are exacerbated in high-stress occupations, where biomarkers of chronic stress show significant elevation (7). Established associations between sleep disorders and increased comorbidity burden—particularly in inflammatory phenotypes of severe insomnia—suggest shared biological mechanisms involving neuroendocrine–immune pathways, which may drive morbidity patterns in radiation workers (7, 11).

Despite extensive research, epidemiological investigations of radiation-exposed cohorts have remained predominantly focused on carcinogenesis, potentially underestimating non-cancer morbidity burdens (12–14). This knowledge gap is particularly salient in southern China, where industrial operations with continuous production cycles and climate-mediated physiological stressors may interact with occupational exposures (12, 14). Preliminary regional studies also suggest significant inter-individual variability in stress biomarker profiles among radiation workers (15, 16). However, a clear gap exists in studies that directly link quantified morbidity burden to sleep outcomes in this population, especially while considering key occupational factors such as radiation exposure dose and work schedules.

This cross-sectional study examines morbidity burden and sleep disorder risk among occupational radiation-exposed workers in southern China. By investigating these relationships within a cohort exposed to both radiation and region-specific co-factors, we aim to characterize comprehensive health challenges beyond oncological endpoints. A key objective is to explore whether morbidity burden serves as a significant correlate of sleep disorders in this unique occupational group, providing a foundation for future research with detailed exposure assessment.

## Methods

### Study design and participants

This cross-sectional study was conducted between January and December 2024 at Guangzhou Twelfth People’s Hospital in southern China. A consecutive sample of 1,089 occupationally radiation-exposed workers undergoing mandatory annual occupational health examinations was recruited.

Inclusion criteria required participants to be: (1) full-time employees with ≥1 year of continuous occupational radiation exposure in medical settings (e.g., diagnostic radiology, nuclear medicine, radiation therapy); (2) aged ≥18 years; and (3) capable of completing structured questionnaires and comprehensive clinical evaluations.

Exclusion criteria encompassed: (1) individuals with non-occupational radiation exposure (e.g., nuclear industry workers); (2) incomplete demographic or health records; and (3) severe cognitive impairment affecting questionnaire validity.

The study protocol received approval from the Ethics Committee of Guangzhou Twelfth People’s Hospital (Approval No. 2023056). Written informed consent was obtained from all participants prior to data collection. Data acquisition integrated electronic occupational health records with face-to-face interviews to verify occupational histories and clinical parameters. The data collection process followed a standardized protocol: (1) eligibility screening using institutional records; (2) informed consent acquisition; (3) administration of the structured questionnaire by trained occupational physicians in a private clinical setting; (4) verification and abstraction of clinical diagnoses from health records; and (5) data entry into a secure electronic database with double-checking for accuracy.

### Sample size

Sample size determination followed pragmatic constraints of annual health surveillance coverage, targeting all eligible workers during the study period. Sample size calculation was performed *a priori* using the formula for cross-sectional studies with a binary outcome variable (sleep disorder prevalence):


$$n=\frac{{Z^{2}}_{1-\alpha /2}\times p\times (1-p)}{\varepsilon^{2}}$$

where:

- Z_1-α/2_ = 1.96 (Z-score for 95% confidence level, α = 0.05).
- *p* = 0.28 (expected sleep disorder prevalence, estimated from pilot data and regional occupational health reports).
- *ε* = 0.03 (margin of error).

This yielded a minimum sample size of 848 participants. To account for potential non-response and missing data (~10% attrition), the target sample size was adjusted to 943 participants.

### Questionnaire on the occupational health status of radiation workers

A questionnaire was developed to systematically assess demographic, occupational, and health-related variables among radiation workers in southern China. Demographic variables included age, sex, height, weight, body mass index (BMI), ethnicity, and education level (categorized as Bachelor’s degree or below, Master’s degree, or PhD). Occupational exposure metrics encompassed years of radiological service, daily working hours, profession (clinical healthcare vs. biomedical engineering), position responsibilities (e.g., diagnostic radiology, interventional radiology), academic title, and in-room operation status. Lifestyle factors were evaluated using standardized categorical scales: smoking status (never smoked, quit, currently smoke), alcohol consumption (never, quit, currently drink), and physical exercise frequency (never, 1–3 times/week, 3–6 times/week, daily). Sleep-related parameters comprised self-reported sleep duration (≤6, 6–8, or ≥8 h/day) and physician-diagnosed sleep disorders (binary yes/no variable).

### Assessment of sleep disorders

Sleep disorders were assessed using a study-specific questionnaire. This decision was based on the need to efficiently integrate sleep assessment into the broader occupational health survey administered during mandatory physical examinations. The questionnaire included key items on sleep quality (rated on a 5-point Likert scale), difficulty falling asleep (taking >30 min), difficulty maintaining sleep (waking up ≥3 times per night), and daytime dysfunction due to sleep issues (e.g., fatigue, concentration problems). A binary outcome (yes/no) for the presence of a sleep disorder was determined based on physician evaluation of the responses in the clinical context of the occupational health examination. The use of physician diagnosis incorporating clinical judgment aimed to enhance specificity, though the lack of a validated instrument is acknowledged as a limitation.

### Morbidity burden assessment

Morbidity burden was assessed through physician-confirmed diagnoses of seven conditions, standardized using ICD-10 codes. Thyroid disease (ICD-10: E00-E07) included hypothyroidism (E03.9) and hyperthyroidism (E05.9). Metabolic disease (E70-E90) encompassed type 2 diabetes (E11.9) and dyslipidemia (E78.5). Respiratory disease (J00-J99) covered pneumonia (J18.9) and chronic obstructive pulmonary disease (J44.9). Digestive disease (K00-K93) included gastritis (K29.7) and gastrointestinal hemorrhage (K92.2). Cardiovascular disease (I00-I99) focused on hypertension (I10) and ischemic heart disease (I25.1). Lens opacity (H25-H28) was defined by age-related cataracts (H25.9). Tumors (C00-D49) included benign (D36.9) and malignant neoplasms.

### Statistical analysis

All analyses were performed using R version 4.3.2 (R Foundation for Statistical Computing). Continuous variables were summarized as mean ± standard deviation and compared using Student’s t-tests. Categorical variables were presented as counts (percentages) and analyzed with Pearson’s chi-squared tests or Fisher’s exact tests, where appropriate. Multivariable logistic regression models were constructed to examine the association between morbidity burden and sleep disorder risk. Three sequential models were developed: crude model (unadjusted), model 1 (adjusted for age and sex), and model 2 (further adjusted for ethnicity, BMI, years of radiological service, daily working hours, education background, professions, position responsibilities, academic title, in-room operation, active smoking status, alcohol consumption status, and physical exercise status). To address potential subgroup instability, interaction terms between morbidity burden and key subgroup variables (sex, years of radiological service, profession) were included in the models to test for effect modification. Additionally, linear trend tests across morbidity categories were performed to assess dose–response relationships. Results were presented as odds ratios (ORs) with corresponding 95% confidence intervals. Subgroup analyses were conducted by stratifying participants according to sex, occupational duration, and profession. Forest plots were generated using the forestplot package to visualize the stratified odds ratios and their precision. All statistical tests were two-tailed, with *p*-values <0.05 considered statistically significant.

## Results

### Characteristics of study participants

The study included 1,089 occupationally radiation-exposed workers. Of these, 359 (33.0%) reported having a sleep disorder, while 730 (67.0%) did not. Overall, the cohort had a mean age of 38.7 ± 9.0 years and comprised 444 men (40.8%) and 645 women (59.2%). Participants with sleep disorders were significantly older (39.7 ± 9.3 vs. 38.2 ± 8.9 years, *p* = 0.009) and more likely to be male (51.8% vs. 35.3%, *p* < 0.001) compared with those without sleep disorders. Educational background showed a strong association with sleep disorder status (*p* < 0.001), with a higher prevalence (67.7%) among those holding a Bachelor’s degree or below compared to Master’s (24.8%) or PhD (7.5%) degrees. Duration of radiological service was marginally longer in those with sleep disorders (11.9 ± 9.1 years vs. 10.7 ± 8.6 years, *p* = 0.036). Differences in daily working hours (*p* = 0.676), ethnicity (*p* = 0.732), professions (*p* = 0.493), position responsibilities (*p* = 0.193), academic title (*p* = 0.622), in-room operation (*p* = 0.532), smoking (*p* = 0.706), and alcohol consumption (*p* = 0.308) were not statistically significant. Critically, self-reported sleep duration of ≤6 h/day was significantly more prevalent in the sleep disorder group (24.2% vs. 12.9%, *p* < 0.001). Detailed information is presented in Table 1.

**Table 1:** Characteristics of eligible participants.

Variables	Overall (*n* = 1,089)	With sleep disorder (*n* = 359)	Without sleep disorder (*n* = 730)	*p*-value
Age/years	38.7 ± 9.0	39.7 ± 9.3	38.2 ± 8.9	**0.009**
Sex				**< 0.001**
Male	444 (40.8)	186 (51.8)	258 (35.3)	
Female	645 (59.2)	173 (48.2)	472 (64.7)	
Ethnicity				0.732
Han	1,067 (98.0)	351 (97.8)	716 (98.1)	
Other	22 (2.0)	8 (2.2)	14 (1.9)	
Height/cm	166.8 ± 8.0	165.4 ± 7.9	167.4 ± 8.1	**< 0.001**
Weight/kg	64.2 ± 11.4	62.6 ± 11.0	64.9 ± 11.6	**0.001**
BMI	23.0 ± 3.1	22.8 ± 2.9	23.1 ± 3.2	0.121
Years of radiological service/years	11.1 ± 8.8	11.9 ± 9.1	10.7 ± 8.6	**0.036**
Daily working hours/h	7.8 ± 1.8	7.8 ± 1.6	7.8 ± 1.9	0.676
Education background				**< 0.001**
Bachelor’s degree and below	645 (59.2)	243 (67.7)	402 (55.1)	
Master’s degree	323 (29.7)	89 (24.8)	234 (32.1)	
Doctor of Philosophy	121 (11.1)	27 (7.5)	94 (12.9)	
Professions				0.493
Clinical healthcare	728 (66.9)	245 (68.2)	483 (66.2)	
Biomedical engineering	361 (33.1)	114 (31.8)	247 (33.8)	
Position responsibilities				0.193
Diagnostic radiology	523 (48.0)	180 (50.1)	343 (47.0)	
Interventional radiology	301 (27.6)	84 (23.4)	217 (29.7)	
Radiation therapy	103 (9.5)	33 (9.2)	70 (9.6)	
Nuclear medicine	69 (6.3)	26 (7.2)	43 (5.9)	
Others	93 (8.5)	36 (10.0)	57 (7.8)	
Academic title				0.622
Junior	381 (35.0)	127 (35.4)	254 (34.8)	
Intermediate	455 (41.8)	155 (43.2)	300 (41.1)	
Senior	253 (23.2)	77 (21.4)	176 (24.1)	
In-room operation	354 (32.5)	122 (34.0)	232 (31.8)	0.532
Active smoking status				0.706
Never smoked	984 (90.4)	328 (91.4)	656 (89.9)	
Have quit smoking	25 (2.3)	8 (2.2)	17 (2.3)	
Currently smoke	80 (7.3)	23 (6.4)	57 (7.8)	
Alcohol consumption status				0.308
Never drink alcohol	543 (49.9)	187 (52.1)	356 (48.8)	
Have quit drinking	13 (1.2)	6 (1.7)	7 (1.0)	
Currently drink alcohol	533 (48.9)	166 (46.2)	367 (50.3)	
Physical exercise status				0.096
Never exercise	199 (18.3)	76 (21.2)	123 (16.8)	
1–3 times/week	738 (67.8)	233 (64.9)	505 (69.2)	
3–6 times/week	114 (10.5)	42 (11.7)	72 (9.9)	
Daily exercise	38 (3.5)	8 (2.2)	30 (4.1)	
Sleep duration				**< 0.001**
≤6 h/day	181 (16.6)	87 (24.2)	94 (12.9)	
6–8 h/day	859 (78.9)	262 (73.0)	597 (81.8)	
≥8 h/day	49 (4.5)	10 (2.8)	39 (5.3)	

Bold values indicate statistically significant *p*-values (<0.05).

### Comparative analysis of disease prevalence between groups

Workers with sleep disorders exhibited substantially higher prevalence of multiple morbidities compared to those without sleep disorders. Thyroid disease occurred in 32.0% of affected workers vs. 19.3% in unaffected workers (*p* < 0.001). Respiratory disease prevalence doubled among those with sleep disorders (22.0% vs. 11.1%, *p* < 0.001), while digestive disease incidence showed an 18.4% vs. 9.0% differential (*p* < 0.001). Cardiovascular conditions demonstrated the starkest contrast (10.3% vs. 2.6%, *p* < 0.001). Lens opacity rates were more than twice among sleep-impaired workers (7.5% vs. 2.7%, *p* < 0.001). Metabolic diseases and tumors showed no significant between-group variation. Detailed information is presented in Table 2.

**Table 2:** Comparative analysis of disease prevalence among occupational radiation workers with and without sleep disorders.

Morbidity	Overall (*n* = 1,089)	With sleep disorder (*n* = 359)	Without sleep disorder (*n* = 730)	*p*-value
Thyroid disease	256 (23.5)	115 (32.0)	141 (19.3)	**<0.001**
Metabolic disease	161 (14.8)	62 (17.3)	99 (13.6)	0.105
Respiratory disease	160 (14.7)	79 (22.0)	81 (11.1)	**<0.001**
Digestive disease	132 (12.1)	66 (18.4)	66 (9.0)	**<0.001**
Cardiovascular disease	56 (5.1)	37 (10.3)	19 (2.6)	**<0.001**
Lens opacity	47 (4.3)	27 (7.5)	20 (2.7)	**<0.001**
Tumor	34 (3.1)	15 (4.2)	19 (2.6)	0.160

Bold values indicate statistically significant *p*-values (<0.05).

### Sleep disorder prevalence with morbidity burden

**Figure 1:**
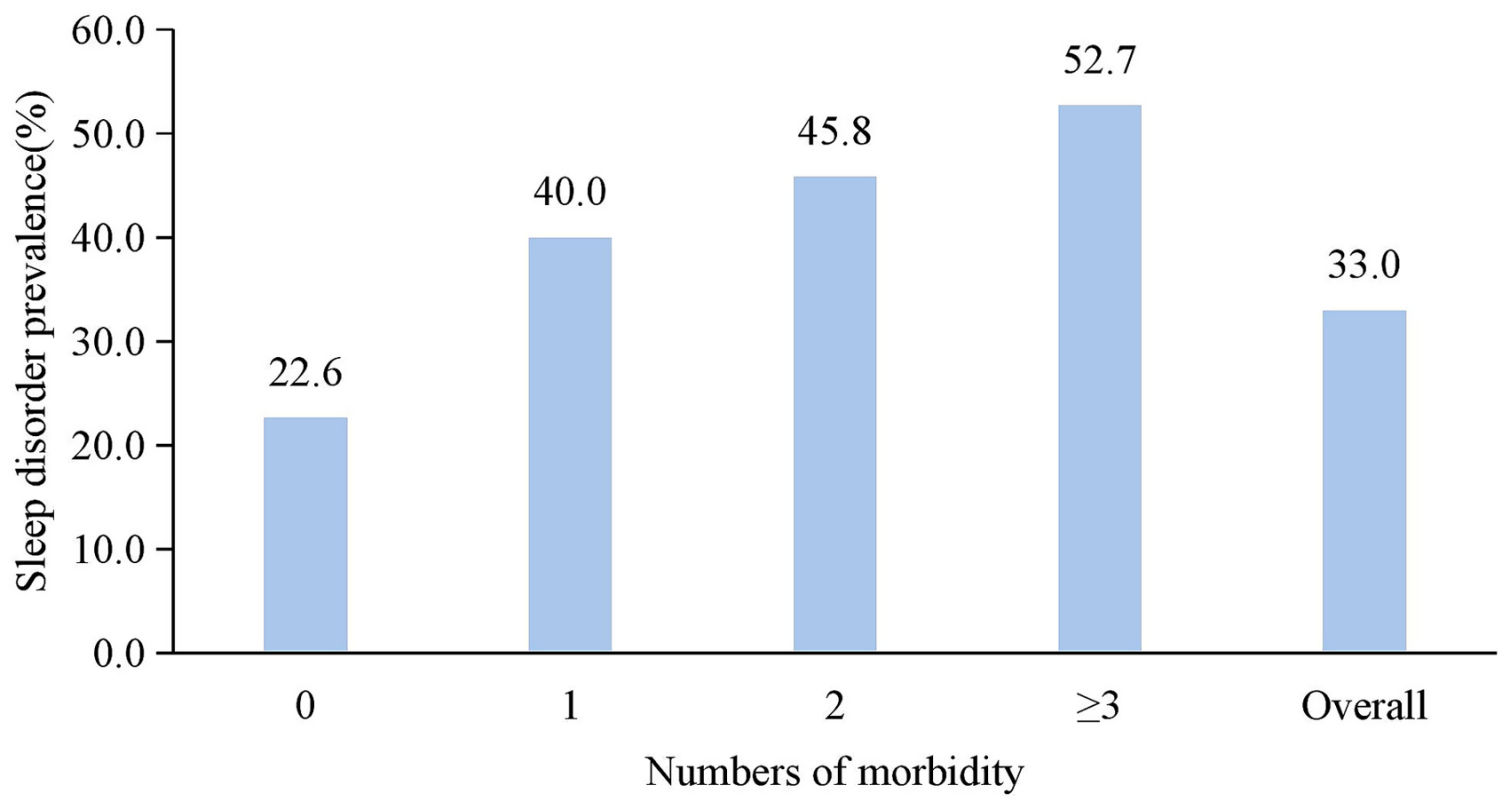
Increasing sleep disorder prevalence with morbidity burden in radiation workers.

Prevalence demonstrated a clear dose-dependent relationship with morbidity count. Workers with no comorbidity exhibited 22.6% prevalence. Those reporting one morbidity showed 40.0% prevalence—nearly double the rate observed in morbidity-free counterparts. Prevalence further increased to 45.8% among workers with two comorbidities. The highest burden group (≥3 comorbidities) displayed a 52.7% prevalence rate, exceeding double the lowest category’s rate. Critically, each incremental morbidity category showed progressively higher sleep disorder frequency compared to the reference (0 comorbidity), as shown in Figure 1.

### Association between morbidity burden and sleep disorder risk

A dose–response relationship existed between morbidity burden and sleep disorder risk (Table 3). Workers with one morbidity exhibited significantly higher sleep disorder prevalence (40.0% vs. 22.6%) and crude risk (OR = 2.28, 95%CI:1.68–3.10) compared with those without comorbidity. This gradient intensified with increasing morbidity counts: workers with two morbidities had 45.8% prevalence (OR = 2.89, 95%CI:1.97–4.25), while those with ≥3 morbidities showed the highest prevalence (52.7%) and risk elevation (OR = 3.81, 95%CI:2.42–6.01). The associations remained statistically significant (all *p* < 0.001) after adjusting for demographic and occupational confounders in Model 1 (age, sex) and Model 2 (additional factors).

**Table 3:** Association between morbidity burden and sleep disorder risk in radiation workers.

Numbers of morbidity	With sleep disorder	Without sleep disorder	Crude model		Adjusted model 1		Adjusted model 2	
			OR (95%CI)	*p*-value	OR (95%CI)	*p*-value	OR (95%CI)	*p*-value
0 (*n* = 557)	126 (22.6)	431 (77.4)	Ref.		Ref.		Ref.	
1 (*n* = 295)	118 (40.0)	177 (60.0)	2.28 (1.68 ~ 3.10)	<0.001	2.23 (1.63 ~ 3.04)	<0.001	2.26 (1.65 ~ 3.09)	<0.001
2 (*n* = 144)	66 (45.8)	78 (54.2)	2.89 (1.97 ~ 4.25)	<0.001	2.78 (1.88 ~ 4.12)	<0.001	2.69 (1.80 ~ 4.02)	<0.001
≥3 (*n* = 93)	49 (52.7)	44 (47.3)	3.81 (2.42 ~ 6.01)	<0.001	3.39 (2.11 ~ 5.45)	<0.001	3.00 (1.85 ~ 4.89)	<0.001

Adjusted model 1: adjusted for age and sex.

Adjusted model 2: adjusted for age, sex, ethnicity, BMI, years of radiological service, daily working hours, education background, professions, position responsibilities, academic title, in-room operation, active smoking status, alcohol consumption status, and physical exercise status.

### Subgroup analysis results

**Figure 2:**
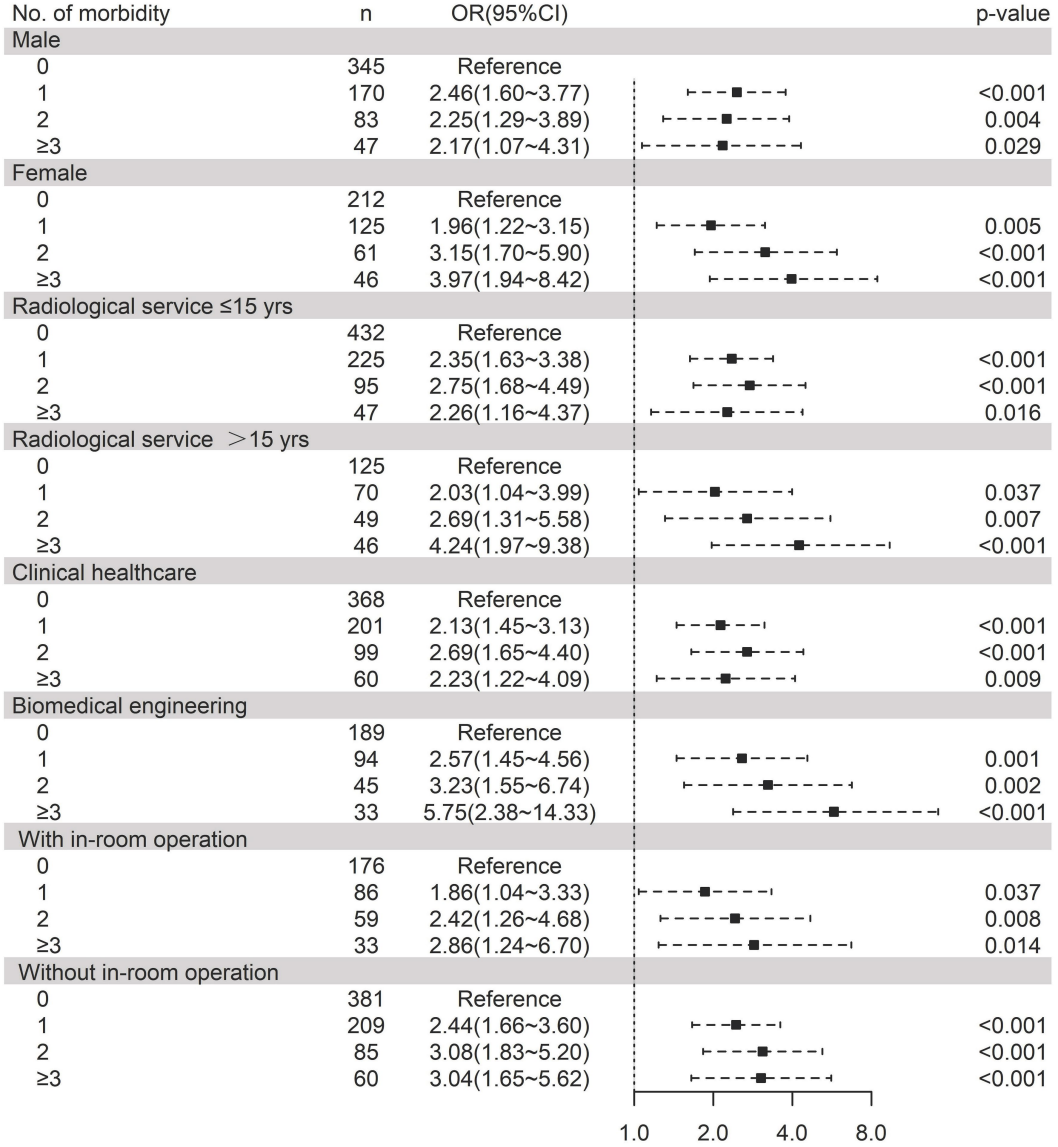
Forest plot of the association between morbidity burden and sleep disorder risk in radiation workers after adjusting for a broad of risk factors.

Stratification by sex revealed markedly stronger associations in women (≥3 morbidity: OR = 3.97, 1.94–8.42) than men (OR = 2.17, 1.07–4.31). Workers with ≤15 years of radiological service exhibited substantially elevated risk (OR = 4.24, 1.97–9.38) vs. >15 years’ experience (OR = 2.69, 1.31–5.58). Biomedical engineers demonstrated exceptional vulnerability (OR = 5.75, 2.38–14.33), contrasting with clinical staff who maintained significant but moderate associations (OR range = 2.23–2.69). The morbidity–sleep disorder link persisted across operational settings, with both in-room (OR = 2.86, 1.24–6.70) and non-in-room workers (OR = 3.04, 1.65–5.62) showing clinically important risks at the highest morbidity levels (Figure 2). However, these subgroup effects should be interpreted with caution due to potential instability from small sample sizes in some strata and multiple comparisons. Formal interaction testing did not reveal statistically significant interactions (sex-by-morbidity interaction *p* = 0.15, profession-by-morbidity interaction *p* = 0.22), suggesting that the observed differences may not be robust. Trend analyses confirmed a significant positive association between increasing morbidity burden and sleep disorder risk across all subgroups (*p*-trend <0.001).

## Discussion

This cross-sectional investigation among 1,089 occupationally radiation-exposed workers in southern China identifies a robust, graded relationship between accumulating physical morbidity burden and elevated sleep disorder risk. Our finding of a 33.0% prevalence of sleep disorders is higher than the reported prevalence in the general adult population in China (approximately 15–20% based on recent meta-analyses) (17, 18), but comparable to rates observed in other high-stress occupational groups such as healthcare workers and industrial shift workers (19, 20). This suggests that occupational stressors, which may include but are not limited to radiation exposure concerns, contribute to the elevated risk.

The dose-dependent escalation in sleep disorder prevalence—22.6% with zero morbidity to 52.7% among those with ≥3 conditions—demonstrates a disease burden effect exceeding additive risk models. Adjusted analysis confirms this gradient persists independently of demographic covariates (OR = 3.81 for the highest morbidity burden), suggesting pathophysiological synergies rather than mere coincidence. The strength of association parallels findings in non-radiation cohorts where multi-morbidity predicts sleep disruption in population studies (21–23). While general pathways involving inflammatory cross-talk between somatic diseases and sleep-regulating neurocircuitry provide a plausible framework—where elevated proinflammatory cytokines (IL-6, TNF-*α*) may disrupt hypothalamic orexin pathways (24)—the specific mechanistic links to occupational radiation exposure remain hypothetical. It is plausible that chronic low-dose radiation may potentiate systemic inflammation or directly affect central nervous system function, but our study lacks direct biological evidence to confirm these mechanisms. Future research integrating biomarkers of radiation effect and neuroinflammation is needed to elucidate the precise biological pathways.

The pronounced gender disparity—near-doubled effect magnitude in women (OR = 3.97) vs. men (OR = 2.17) at the highest morbidity burden—demands biological and psychosocial consideration. Women exhibit greater susceptibility to inflammation-mediated disorders (23), potentially amplifying the cytokine-sleep disturbance cascade. Furthermore, sex-based variations in hypothalamic–pituitary–adrenal axis reactivity to chronic stressors (25) likely interact with occupational radiation anxiety. Although radiation-specific anxiety might contribute, the current evidence does not allow us to distinguish this from general occupational stress effects. This finding extends observations from nuclear medicine staff where women report higher psychosomatic symptom burdens despite equivalent radiation exposure metrics (26).

Occupational parameters yielded unexpected insights. Workers with ≤15 years’ service exhibited stronger morbidity–sleep disorder associations (OR = 4.24) than veterans (>15 years: OR = 2.69). This contradicts conventional duration–risk assumptions but aligns with vigilance-decrement models, where heightened anxiety during early career phases potentiates stress responses (27). Biological adaptation through epigenetic regulation of stress–response genes over prolonged low-dose exposure represents one plausible hypothesis (28), although this remains speculative without direct mechanistic evidence. Strikingly, biomedical engineers manifested exceptional vulnerability (OR = 5.75 at highest morbidity burden), in contrast to the attenuated risk profile observed in clinical staff. This divergence might originate from differential coping mechanisms—clinical personnel typically receive formal psychosocial support, absent for engineers (29). Additionally, distinct radiation source proximity patterns merit investigation; engineers’ frequent handling of unshielded equipment during maintenance could provoke heightened perceived risk.

The absence of metabolic disease associations diverges substantially from general population data, where diabetes mellitus independently associates with a 2.3-fold increased sleep disorder risk (30). This discordance highlights potential radioprotective mechanisms requiring investigation. Ionizing radiation modifies adipokine secretion patterns (31), possibly altering metabolic disease phenotypes, but this hypothesis requires validation. However, current data cannot exclude underdiagnosis bias given occupational health screenings’ restricted metabolic panels. Contrastingly, the thyroid disorder prevalence gap (32.0% vs. 19.3% between sleep-impaired and unaffected workers) echoes documented radiation sensitivity of thyrocytes (32). Radioiodine accumulation disproportionately disrupts circadian-regulated thyroid hormone conversion, creating pathogenic synergy with sleep dysregulation (4)—this represents one of the more direct biological pathways potentially linking radiation exposure to sleep outcomes in our cohort.

Notably, self-reported short sleep duration (≤6 h) prevalence nearly doubled among workers with sleep disorders (24.2% vs. 12.9%). This finding is particularly relevant in the radiation context, as experimental evidence demonstrates that sleep restriction amplifies radiation-induced oxidative stress markers (9, 15). Short sleep duration fundamentally impairs DNA double-strand break repair kinetics (33), potentially establishing a unique bidirectional pathology among radiation workers. The observed epidemiological relationship likely understates the true biological effect magnitude due to underreporting of sleep problems in occupational settings (34).

Several limitations of this study must be acknowledged. First, the cross-sectional design precludes causal inference. Second, the lack of personal radiation dosimetry data is a critical limitation, as it prevents us from examining dose–response relationships with radiation exposure itself, which is a core occupational exposure in this cohort. Our findings therefore primarily speak to the association between morbidity burden and sleep disorders within a group defined by occupational radiation exposure, rather than establishing a direct link with radiation dose (22–24). Future studies incorporating cumulative dose metrics are essential. Third, the use of a study-specific questionnaire for sleep assessment, rather than a validated instrument like the Pittsburgh Sleep Quality Index (PSQI) or Insomnia Severity Index (ISI), limits the comparability of our prevalence estimates with other studies and may have introduced measurement error. Fourth, we did not assess important potential confounders such as psychological distress (e.g., anxiety, depression) or detailed shift work patterns (e.g., specific rotation schedules) in the primary analysis (35), which could bias the observed associations. Although we adjusted for several lifestyle factors, residual confounding remains possible. The subgroup findings, particularly for professions with small numbers, should be interpreted cautiously as they may be unstable. Finally, the generalizability of our findings to radiation workers in other regions or with different occupational profiles may be limited.

Notwithstanding these limitations, the study has notable strengths, including a substantial sample size, a comprehensive assessment of morbidity based on physician diagnoses, and adjustment for a wide range of demographic and occupational factors.

## Conclusion

Accumulating morbidity burden demonstrates a robust, occupationally modulated association with sleep disorder risk in radiation workers. However, the study’s limitations, particularly the lack of quantitative radiation exposure data and the cross-sectional nature, necessitate that these findings be considered exploratory. The elevated prevalence of sleep disorders underscores the need for integrating sleep health into occupational health protocols for this workforce. Biomedical engineers, women, and early-career personnel emerged as subgroups with numerically higher point estimates, potentially warranting closer attention in clinical practice, though these differences were not statistically significant in interaction tests. Future longitudinal studies incorporating quantitative radiation dosimetry, validated sleep measures, psychological assessments, and precise shift work history are crucial to confirm these associations and elucidate the underlying causal pathways.

## Data Availability

The raw data supporting the conclusion of this article will be made available by the authors, without undue reservation.
